# *Cynanchi atrati* and Its Phenolic Constituent Sinapic Acid Target Regulator of Calcineurin 1 (RCAN1) to Control Skin Inflammation

**DOI:** 10.3390/antiox11020205

**Published:** 2022-01-21

**Authors:** Seon Sook Kim, Nam Kyoung Kim, Su Ryeon Seo

**Affiliations:** 1 Department of Molecular Bioscience, College of Biomedical Science, Kangwon National University, Chuncheon 24341, Korea; painniche@kangwon.ac.kr; 2 Institute of Bioscience & Biotechnology, Kangwon National University, Chuncheon 24341, Korea; 3 R&D Center, Medipeau Inc., Chuncheon 24398, Korea; kimnk@medipeau.co.kr

**Keywords:** *Cynanchi atrati*, atopic dermatitis (AD), regulator of calcineurin 1 (RCAN1), NF-κB, inflammation, sinapic acid (SA)

## Abstract

Atopic dermatitis (AD) is a common inflammatory skin disorder, and numerous pharmacological approaches are employed to reduce symptoms. Natural products of plant-derived materials have been accepted as complementary therapy for the treatment of a wide range of inflammatory diseases. *Cynanchi atrati* (CA) is an oriental medicinal herb used in the treatment of acute urinary infection, febrile diseases, and laryngopharyngitis. However, the role of CA root extract in skin inflammation such as AD has not been explored yet. In this study, we examined the possible effect of CA root extract on skin inflammation and evaluated the underlying signaling mechanism using in vitro and in vivo modeling systems. Raw264.7 macrophages were used for in vitro experiments, and an oxazolone-induced AD mouse model was used to evaluate in vivo effects. CA extract significantly inhibited the expression levels of lipopolysaccharide (LPS)-induced pro-inflammatory cytokines such as interleukin-6 (IL-6) and interleukin-1β (IL-1β) in RAW264.7 macrophages. The CA root extract mediated suppression of pro-inflammatory cytokine expression and was associated with the decreased nuclear factor kappa B (NF-κB) gene transcriptional activation. Moreover, CA root extract attenuated the in vivo expression of IL-6 and tumor necrosis factor-α (TNF-α) and ear swelling in the AD mouse models. We also observed that the inhibitory effect of CA root extract on skin inflammation was accompanied by the upregulation of calcineurin 1 (RCAN1) expression, which functions in the inflammatory pathways by suppressing NF-κB signaling. We consistently observed that the immunosuppressive effect of CA root extract in AD was significantly perturbed in the RCAN1 knockout mice. In addition, we isolated a phenolic acid compound, sinapic acid (SA), from the CA root extract and found that SA consistently exerted an immunosuppressive effect in RAW264.7 macrophages by inducing RCAN1 expression. Our results provide the first evidence that CA root extract and its phenolic acid constituent, SA, modulate NF-κB signaling pathways by inducing RCAN1 expression in the skin inflammation process. Thus, we suggest that CA root extract has a therapeutic value for the treatment of AD by targeting endogenous immune regulators.

## 1. Introduction

Atopic dermatitis (AD) is a chronic inflammatory skin disease with severe itching and dried skin, and it usually begins in infancy or childhood [1,2]. Approximately 10–20% of children have atopic dermatitis, and when they become adults, the symptoms usually improve or disappear; however, approximately 1–3% of patients continue to develop symptoms into adulthood [3]. Although the cause of atopic dermatitis has not been clarified, genetic, environmental, and immunological factors have been reported to be involved. In particular, the incidence of atopic dermatitis is increasing worldwide due to environmental factors. In patients with atopic dermatitis, the excessive activation of immune cells is observed. Recently, many studies have been conducted to improve symptoms by suppressing excessive inflammatory reactions in atopic dermatitis. Currently, steroids, anti-histamines, and immunosuppressants are used for the treatment of atopic dermatitis, and each of them may cause serious side effects to the human body, which makes it difficult for continuous use. In addition, most patients with atopic dermatitis are infants and children. Therefore, there is a constant demand for research on new substances that have fewer side effects and can be used without difficulty in infants. One of the hallmarks of AD is the overproduction of pro-inflammatory cytokines such as interleukin-4 (IL-4), IL-6, IL-1β, and TNF-α. These cytokines facilitate the release of various inflammatory chemokines that recruit immune cells to the lesion site of AD.

RCAN1 is a gene located near the Down Syndrome Critical Region (DSCR) of human chromosome 21 [4]. RCAN1 is named according to its function of binding to calcineurin, a calcium/calmodulin-dependent serine/threonine phosphatase [5]. Calcineurin is known to play an important role in the development and maturation of the nervous system, cardiovascular system, and immune system [6]. Several recent reports have shown that RCAN1 is induced by various stimulators and functions as a mediator in the inflammatory signaling pathways [7,8,9]. In endothelial cells, RCAN1 functions to regulate the expression of inflammatory cytokines by inhibiting the nuclear factor of activated T cells (NFAT) family of transcription factors [7]. RCAN1 deficient bone marrow-derived mast cells (BMMC) showed enhanced activation of NFAT and NF-κB signaling [10]. In U87MG cells, RCAN1 leads to the inhibition of NF-κB by increasing the stability of the IκB protein [8]. In contrast to these reports, RCAN1 is required for the activation of allergic lung inflammation [11].

*Cynanchi atrati* is a member of the Asclepiadaceae family and is widely distributed throughout China, Korea, and Japan. It has been used as a traditional medicine to treat fever, urinary tract infection, edema, and rheumatic arthralgia [12]. Previous pharmacological investigations have reported that C. atrati (CA) has cytotoxic, anti-inflammatory, and anti-acetylcholinesterase activity [13,14]. Despite its diverse pharmacological activities, the underlying molecular mechanism remains unclear. 

Sinapic acid (SA, 4-hydroxy-3,5-dimethoxy cinnamic acid) is a phenylpropanoid compound commonly present in natural herbs and has been used in traditional Chinese remedies [15]. SA has been reported to act as an antioxidant and can be used for the treatment of oxidation-related diseases [16]. The antiproliferative effects of SA on T48D breast cancer cells have been reported [17]. SA shows a neuroprotective function in kainic acid-induced hippocampal neuronal damage in mice [18]. SA prevents inflammatory colitis by inhibiting the expression of TNF-α, malondialdehyde (MDA), and myeloperoxidase (MPO) [19]. SA consistently exerts an anti-inflammatory effect on serotonin-induced paw edema in mice [20].

In this study, we investigated the role of CA root extract in inflammatory signaling pathways in the skin using in vitro and in vivo experimental models. We found that CA root extract and its phenolic constituent, SA, effectively exert anti-inflammatory effects by inducing the expression of RCAN1.

## 2. Materials and Methods

### 2.1. Materials

Lipopolysaccharide (LPS), 4-ethoxymethylene-2-phenyl-2-oxazolin-5-one (oxazolone), and anti-RCAN1 antibodies were purchased from Sigma-Aldrich (St. Louis, MO, USA). The anti-IL-1β and anti-phospho-IκB antibodies were purchased from Cell Signaling Technology (Danvers, MA, USA). The anti-GAPDH antibodies were purchased from Santa Cruz Biotechnology (Dallas, TX, USA). The series of RCAN1 shRNA expression vectors was purchased from Origene (Rockville, MD, USA). The reporter gene RCAN1-Luc was previously described [21].

### 2.2. Preparation of the C. atrati Root Extract

The dried *C. atrati* (CA) root was purchased from Daekwang Medicine Company (Chuncheon, Korea), and the extraction procedure was followed as in the previous report [22]. Briefly, CA was powdered and extracted in 70% ethanol at room temperature for 3 h. The filtered extract was evaporated to remove solvent (LABOTORA 4000eco, Heidolph Instruments GmbH&Co., Schwabach, Germany) and stored in a freezer at −20 °C until used. 

For the compositional analysis of CA, sample solution was subjected to ultrahigh pressure liquid chromatography system (UHPLC) using an LTQ Orbitrap XL linear ion trap mass spectrometry (MS) system (Thermo Scientific, San Jose, CA, USA) equipped with an electrospray ionization source. Chromatographic separation was performed using an ACQUITY UPLC^®^ BEH C_18_ column (2.1 × 150 mm, 1.7 μm; Waters, Milford, MA, USA) and operated using mobile phases A (0.1% formic acid in water) and B (0.1% formic acid in acetonitrile). Each compound was detected with photodiode array at 200~500 nm. The Orbitrap analyzer was used for high-resolution and accurate mass (HR/AM) data acquisition with 30,000 FWHM resolving power at 400 m/z. The MS/MS experiments were adjusted using the Xcalibur system (Thermo Scientific, San Jose, CA, USA) and performed under automatic gain control (AGC) conditions.

### 2.3. Cell Culture 

The murine RAW264.7 macrophage cells were obtained from ATCC (Manassas, VA, USA). The cells were maintained in DMEM supplemented with 10% fetal bovine serum (FBS), penicillin, and streptomycin. Cells were cultured at 37 °C in a humidified 5% CO_2_ incubator. 

### 2.4. Western Blot Analysis

Cells were lysed with 1% Nonidet P-40 lysis buffer in the presence of protease inhibitors [22]. The cell lysates were centrifuged, and the supernatant was separated by SDS-PAGE. The separated proteins were transferred to nitrocellulose (NC) membranes. The NC membranes were blocked with 5% non-fat dried milk in 0.05% Tween20 containing TBS buffer (20 mM Tris-Cl, pH 7.6, 137 mM NaCl) for 30 min and incubated overnight with the appropriate antibodies at 4 °C. The bands were visualized with ECL.

### 2.5. Reporter Gene Assay

Cells were plated in a 6-well culture dish and transfected when 80% confluent with 0.5 µg of pNF-κB-luc (*firefly* luciferase reporter) and 0.1 µg of pTK-luc (*renilla* luciferase reporter) using the Lipofectamine 3000 (Invitrogen, Carlsbad, CA, USA). Transfection was performed according to the manufacturer’s instructions. At 24 h following transfection, luciferase activity was measured using the dual luciferase assay system (Promega, Madison, WI, USA). The *firefly* luciferase activity was normalized to the *renilla* luciferase activity system.

### 2.6. RT-PCR

Total RNA was extracted according to the manufacturer’s instructions with 1 mL TRIzol reagent (Invitrogen, Carlsbad, CA, USA). Reverse transcription and PCR were performed as previously described [23]. The primers were as follows: RCAN1 (NM_019466.4), 5′-TGCTTGTGTGGCAAACGATG-3′ (forward) and 5′-AGGAACTCGGTCTTGTGCAG-3′ (reverse); IL-1β (NM_008361.4), 5′-ACCTGTTCTTTGAGGCTGAC-3′ (forward) and 5′-CTTCTTTGGGTATTGTTTGG-3′ (reverse); IL-6 (NM_031168.2), 5′-AGTTGCCTTCTTGGGACTGA-3′ (forward) and 5′-TTCTGCAAGTGCATCATCGT-3′ (reverse); TNF-α (NM_013693.3), 5′-TAGCCCACGTCGTAGCAAAC-3′ (forward) and 5′-GGAGGCTGACTTTCTCCTGG-3′ (reverse); β-actin (NM_007393.5), 5′-CATGTTTGAGACCTTCAACACCCC-3′ (forward) and 5′-GCCATCTCTTGCTCGAAGTCTAG-3′ (reverse). Band intensities were quantified using a Gel Logic 100 imaging system (Kodak, Japan).

### 2.7. Oxazolone-Induced Atopic Dermatitis (AD) Model

ICR mice were purchased from Doo Yeol Biotech (Seoul, Republic of Korea), and RCAN1-KO mice were generously provided by Dr. Kwan-Hyuck Baek [24]. Mice were housed in a light-, temperature-, and humidity-controlled breeding facility at the Animal Center of Kangwon National University and provided standard chow and water. The Institutional Animal Care and Use Committee (IACUC) of Kangwon National University approved all the experimental procedures (KW-151002-1). Eight-week-old mice were sensitized with 20 μL of 1.5% oxazolone solution dissolved in a mixture of acetone and olive oil (3:1) to the inner and outer surface of the ears. After one week of sensitization, 20 μL of 0.3% oxazolone solution was repeatedly applied to the ears for an additional week at 2-day intervals. Twenty microliters of CA extract (10 μg/mL) or vehicle (DMSO) was applied daily from day 7 to day 14. Ear thickness was measured using a digital caliper (Mitutoyo, Japan) every day. On day 15, the mice were sacrificed, and ear tissues were collected for further analysis. The number of mice in each group was 5–7.

### 2.8. Histology

Isolated tissues were fixed with 4% formalin and embedded in paraffin blocks. The blocks were sectioned (2–3 μm) and stained with hematoxylin and eosin (H&E). Histopathological changes were examined by light microscopy (Olympus, Tokyo, Japan) and photographed.

### 2.9. Statistical Analysis

Densitometric scans of the Western blot and RT-PCR were quantified using ImageJ software (NIH, Bethesda, MD, USA). The statistical analysis was performed with SPSS (IBM, Armonk, NY, USA). The mean values between the two groups were analyzed using Student’s *t*-test. Comparisons between multiple groups were completed by ANOVA followed by Bonferroni post hoc tests, where *p* < 0.05 was considered statistically significant.

## 3. Results

### 3.1. CA Extract Inhibits the Expression of Pro-Inflammatory Cytokines

**Figure 1:**
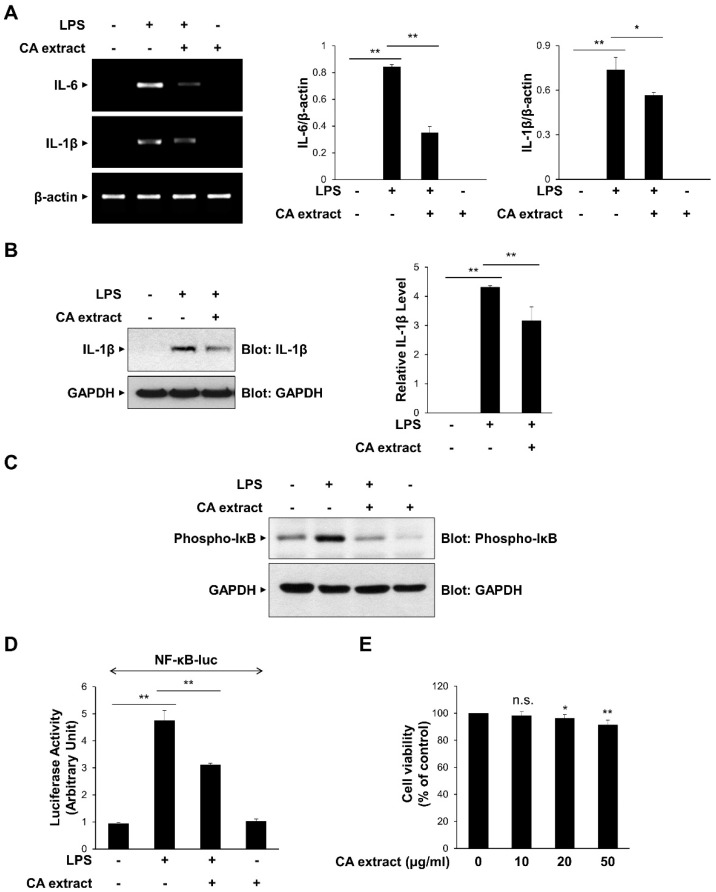
Inhibitory effects of *C. atrati* (CA) extract on the LPS-induced pro-inflammatory cytokine expression. RAW264.7 cells were treated with the CA extract (10 μg/mL) for 30 min before LPS (10 ng/mL) treatment for 3 h. (**A**) The mRNA levels of IL-6, IL-1β, and β-actin were measured by RT-PCR and then quantified. (**B**) The cell lysates were immunoblotted with anti-IL-1β and anti-GAPDH antibodies and then quantified. (**C**) RAW264.7 cells were pretreated with the CA extract (10 μg/mL) for 30 min before LPS (10 ng/mL) treatment for 3 h. The cell lysates were immunoblotted with anti-phospho-IκB and anti-GAPDH antibodies. (**D**) RAW264.7 cells were transfected with NF-κB-luciferase reporter vector. After 24 h, cells were pretreated with the CA extract (10 μg/mL) for 30 min before LPS (10 ng/mL) treatment for 3 h, and cell lysates were analyzed for luciferase activity. (**E**) RAW264.7 cells were treated with the CA extract at the indicated concentrations. After 24 h, cell viability was analyzed by MTT assay. The graphs are presented as the mean ± SD of the three independent experiments. (**A**,**B**,**D**) * *p* < 0.05, ** *p* < 0.01 compared with indicated group. (**E**) n.s (*p* > 0.05), * *p* < 0.05, ** *p* < 0.01 compared with vehicle group.

To determine the effect of CA extract on inflammatory signaling pathways, we first examined the effects of 10 μg/mL CA on the LPS-triggered IL-6 and IL-1β mRNA expressions in RAW264.7 macrophages. As shown in Figure 1A, IL-6 and IL-1β mRNA expression levels after LPS treatment were significantly inhibited by CA pretreatment. The inhibitory effect of CA on pro-inflammatory cytokine expression was consistently observed at the protein level of IL-1β (Figure 1B). We also examined the effect of CA treatment on the transcriptional activation of NF-κB, which is known to be involved in the expression of cytokines [25,26]. We measured IκB phosphorylation, which subsequently activates NF-κB, using a phospho-specific antibody. CA extract effectively inhibited IκB phosphorylation in RAW264.7 cells following LPS stimulation (Figure 1C). The inhibitory effect of CA extract on LPS-induced NF-κB gene transcriptional activation using a reporter assay was consistently observed (Figure 1D). To determine whether CA extract could induce cytotoxicity at our experimental concentrations, we used the MTT assay to measure cell viability after culturing RAW264.7 cells for 24 h in the presence of 10, 20, and 50 μg/mL CA extract (Figure 1E). CA extract did not induce cytotoxic effects in RAW264.7 cells at 10 μg/mL up to 24 h, indicating that the inhibition of cytokine expression was not caused by cell cytotoxicity. Collectively, these results suggest that CA extract inhibits LPS-induced pro-inflammatory cytokine expression by inhibiting the NF-κB signaling pathway. 

### 3.2. CA Extract Suppresses the Inflammatory Response in an Oxazolone-Induced AD Mouse Model

**Figure 2:**
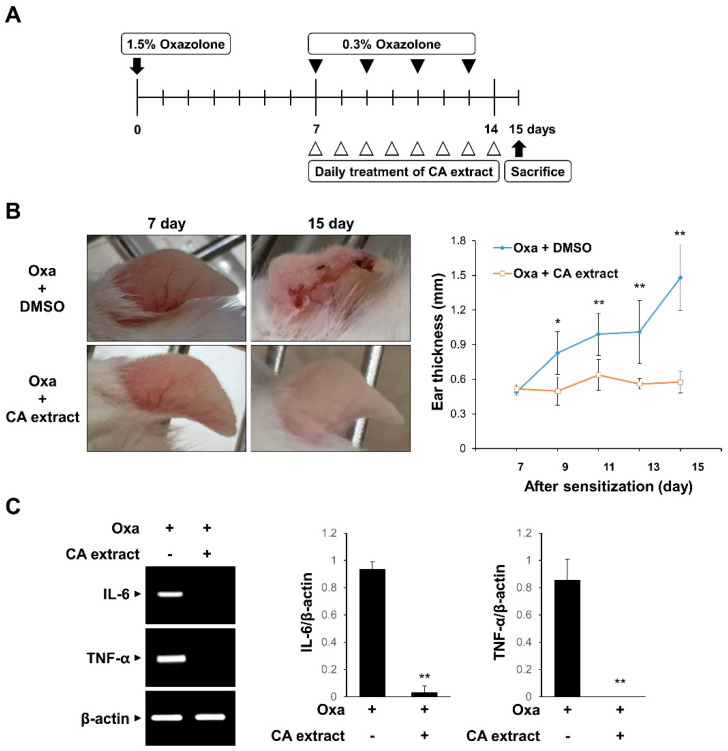
Inhibitory effects of CA extract in a mouse model of oxazolone-induced atopic dermatitis (AD). (**A**) Schematic representation of the experiment. (**B**) Two groups of female ICR mice (*n* = 3 per group) were treated with oxazolone together with either vehicle (DMSO) or CA extract. Imaging analysis was performed on days 7 and 15. The extent of ear edema was assessed by measuring ear thickness at the indicated intervals using a micrometer. Data represent the mean ± SD (*n* = 5). (**C**) mRNA levels of IL-6, TNF-α, and β-actin of ear tissues were measured using RT-PCR and then quantified. * *p* < 0.05, ** *p* < 0.01 compared with oxazolone-treated group.

To evaluate the in vivo effect of CA extract on inflammation, we examined the effect of CA in a mouse model of oxazolone-induced AD. The experimental scheme is shown in Figure 2A. The oxazolone-challenged group showed erythema, crusting, and excoriation, which are typical symptoms of AD (Figure 2B). However, such skin lesions were significantly reduced in the CA extract-treated groups (Figure 2B). In agreement with the phenotypic observations, CA treatment decreased the ear thickness, a marker for the extent of edema, in response to oxazolone-induced AD (Figure 2B). We also determined the mRNA levels of pro-inflammatory cytokines, including IL-6 and TNF-α, in ear tissues using RT-PCR. As shown in Figure 2C, IL-6 and TNF-α mRNA levels were completely blocked by the CA extract treatment. These results suggest that the CA extract attenuates skin inflammation in oxazolone-induced AD mice.

### 3.3. CA Extract-Induced RCAN1 Expression Inhibits LPS-Triggered NF-κB Signaling

**Figure 3:**
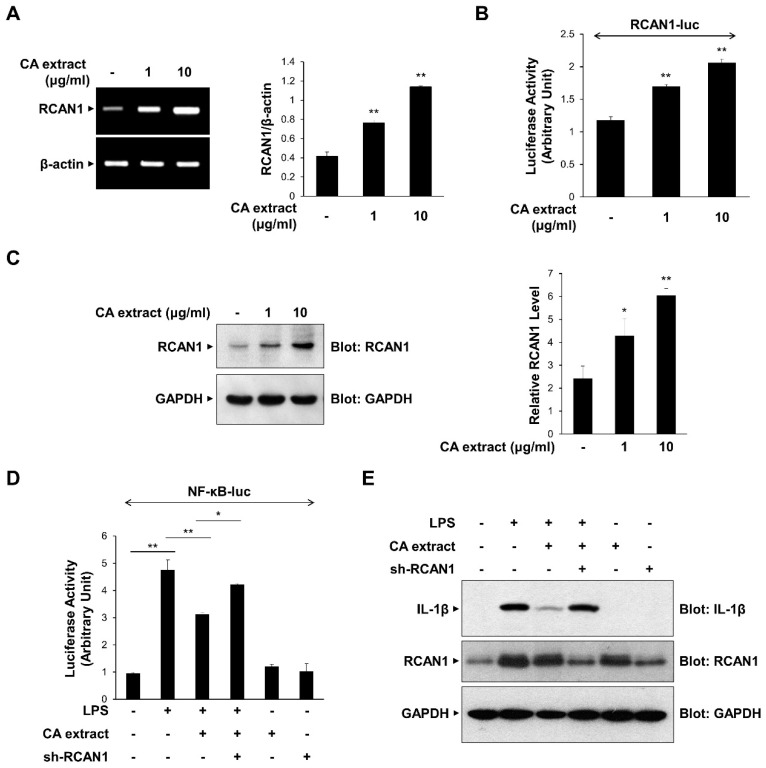
Upregulation of RCAN1 expression in response to CA extract. (**A**) RAW264.7 cells were treated with CA extract at the indicated concentrations. The mRNA levels of RCAN1 and β-actin were measured by RT-PCR and then quantified. (**B**) RAW264.7 cells were transfected with the RCAN1-luciferase reporter vector. After 24 h, cells were treated with the CA extract for 3 h, and cell lysates were analyzed for luciferase activity. (**C**) RAW264.7 cells were treated with CA extract at the indicated concentrations. After 24 h, the cell lysates were immunoblotted with anti-RCAN1 and anti-GAPDH antibodies. The relative levels of RCAN1 were quantified and plotted (*n* = 3). (**D**) RAW264.7 cells were transfected with the NF-κB-luciferase reporter vector alone or together with either the shRNA expression vector or the scrambled RNA expression vector. After 24 h, cells were pretreated with the CA extract (10 μg/mL) for 30 min before LPS (10 ng/mL) treatment for 3 h, and cell lysates were analyzed for luciferase activity. (**E**) RAW264.7 cells were transfected with either the shRNA expression vector or the scrambled RNA expression vector. After 24 h, cells were pretreated with the CA extract (10 μg/mL) for 30 min before LPS (10 ng/mL) treatment for 3 h. The cell lysates were immunoblotted with anti-IL-1β, anti-RCAN1, and anti-GAPDH antibodies. The graphs are presented as the means ± SD of three independent experiments. (**A**–**C**) * *p* < 0.05, ** *p* < 0.01 compared with vehicle group. (**D**) * *p* < 0.05, ** *p* < 0.01 compared with indicated group.

Previous studies have shown that RCAN1 is upregulated by various extracellular signaling molecules and modulates the inflammatory signaling process by inhibiting NF-κB [8,9]. We examined the possible role of RCAN1 in CA-mediated anti-inflammatory signaling mechanisms. We first monitored the effect of CA extract on the expression of RCAN1 mRNA transcript using RT-PCR. As shown in Figure 3A, CA extract (1 and 10 μg/mL) caused an increase in RCAN1 mRNA levels in RAW264.7 cells. To further investigate CA-dependent RCAN1 transcription, we used the RCAN1-reporter construct, which contains a promoter of the RCAN1 gene [21]. CA induced an increase in RCAN1 promoter activity compared with untreated cells (Figure 3B). RCAN1 protein levels are consistently increased by CA treatment, indicating that CA induces RCAN1 expression (Figure 3C). We also examined whether RCAN1 is involved in the CA-mediated anti-inflammatory signaling mechanism. We monitored whether RCAN1 knockdown could prevent the CA-mediated suppression of NF-κB transcriptional activation. As shown in Figure 3D, the knockdown of endogenous RCAN1 attenuated the CA-mediated decrease in NF-κB-luciferase activity. The CA-mediated suppression of IL-1β protein levels was consistently abrogated by RCAN1 knockdown (Figure 3E). Collectively, these results indicate that the reduction in pro-inflammatory cytokine expression by CA extract is dependent on RCAN1 expression.

### 3.4. RCAN1 Is Required for the CA-Mediated Anti-Inflammatory Effects In Vivo

**Figure 4:**
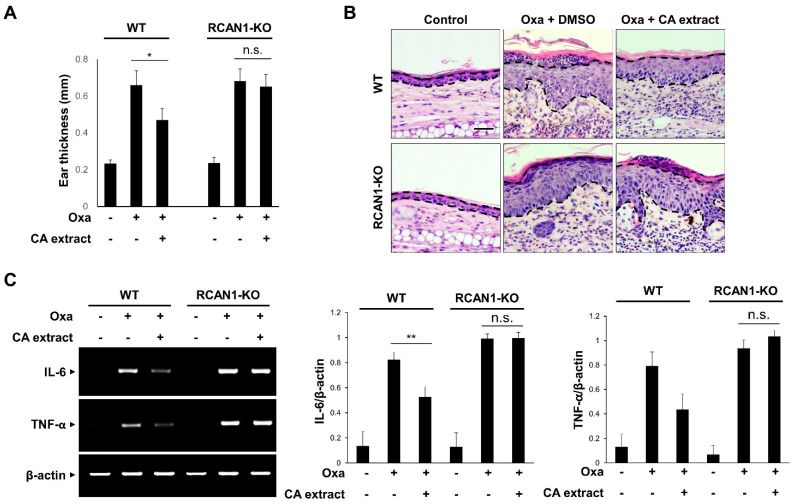
RCAN1-mediated immunosuppression in AD mice. Wild-type (WT) and RCAN1 knockout mice (RCAN1-KO) (*n* = 5–7 per group) were treated with oxazolone together with either vehicle (DMSO) or CA extract (10 μg/mL). (**A**) Ear thickness was measured with a micrometer. Data represent mean ± SD (*n* = 5–7). (**B**) Formalin-fixed ear tissue sections were stained with hematoxylin and eosin (H&E). Bars: 50 μm. (**C**) The mRNA levels of IL-6, TNF-α, and β-actin were measured from ear tissues using RT-PCR and quantified. The graphs are the means ± SD of three independent experiments. n.s. (*p* > 0.05), * *p* < 0.05, ** *p* < 0.01 compared with indicated group.

To verify that RCAN1 is involved in the suppressive action of CA extract on the inflammatory signaling pathway, we examined the effect of CA extract in RCAN1 knockout mice using an AD model. As shown in Figure 4A, the CA-mediated decreased ear thickness in response to oxazolone was significantly perturbed in the RCAN1 knockout mice. To examine the histopathological features of the ear skin lesions, H&E staining analysis was performed. As shown in Figure 4B, the epidermal thickening induced by cell hyperplasia and immune cell infiltration into the ears was reduced in CA-treated AD mice compared with the control mice. However, these CA-mediated ameliorative effects were not observed in the RCAN1 knockout AD mice (Figure 4B). To further characterize the role of RCAN1 in the CA-mediated anti-inflammatory effect, we monitored the mRNA levels of pro-inflammatory cytokines in ear tissues. As shown in Figure 4C, oxazolone-induced IL-6 and TNF-α levels were greatly decreased in the CA-treated wild-type AD mice, whereas CA failed to reduce the level of these cytokines in RCAN1 knockout AD mice. Collectively, these results suggest that the CA-mediated amelioration of AD symptoms occurred in the presence of RCAN1.

### 3.5. Sinapic Acid (SA) Mediates the CA-Induced Anti-Inflammatory Effects

**Figure 5:**
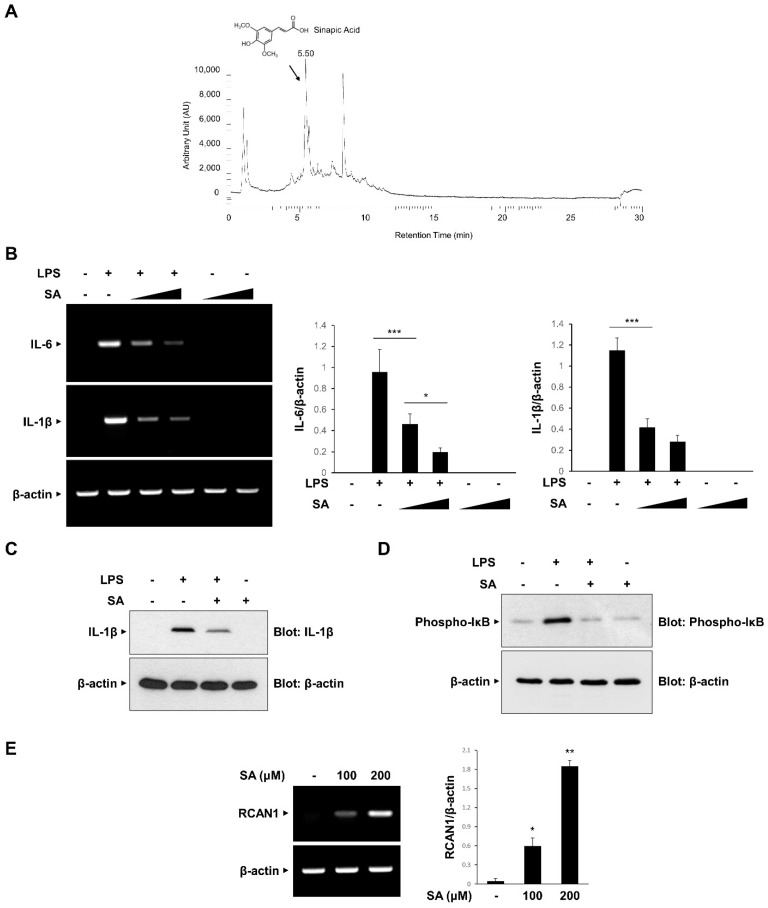
Inhibitory effect of sinapic acid (SA) on the inflammatory signaling pathway. (**A**) Isolation of sinapic acid (SA). (**B**) RAW264.7 cells were treated with the SA (100 and 200 μM) for 30 min before LPS (10 ng/mL) treatment for 3 h. The mRNA levels of IL-6, IL-1β, and β-actin were measured by RT-PCR and then quantified. ∆ represents increasing concentrations of SA. (**C**,**D**) RAW264.7 cells were pretreated with the SA (100 μM) for 30 min before LPS (10 ng/mL) treatment for 3 h. The cell lysates were immunoblotted with anti-IL-1β, anti-phospho-IκB, and anti-GAPDH antibodies. (**E**) RAW264.7 cells were treated with SA at the indicated concentrations. The mRNA levels of RCAN1 and β-actin were measured using RT-PCR and quantified. The graphs are the means ± SD of three independent experiments. (**B**) *** *p* < 0.001 compared with indicated group. (**E**) * *p* < 0.05, ** *p* < 0.01 compared with vehicle group.

**Figure 6:**
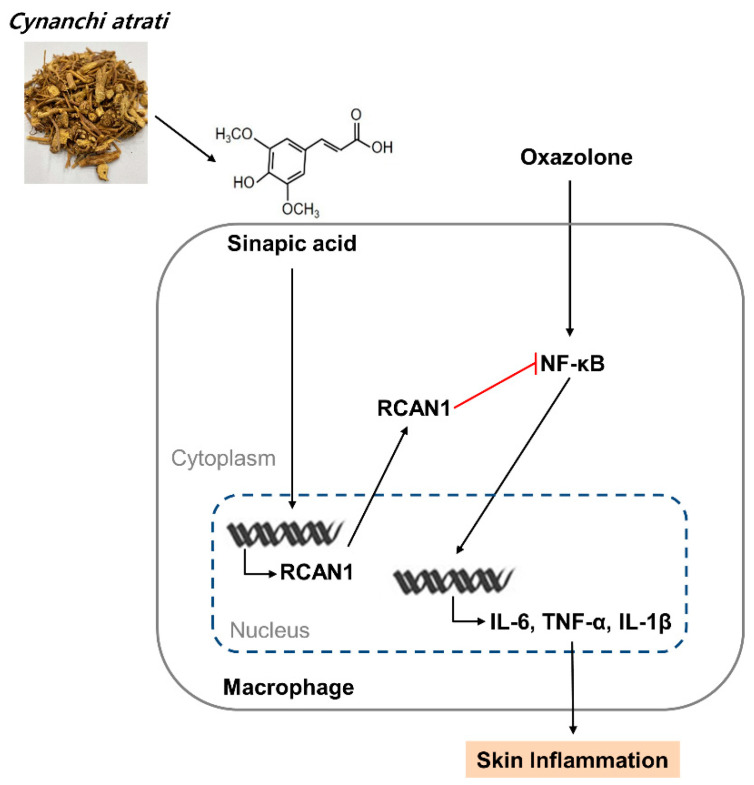
A schematic model of CA extract on the suppression of skin inflammation. CA extract induces RCAN1 expression, which in turn suppresses oxazolone-induced cytokine expressions by inhibiting NF-kB activation in AD mice.

We attempted to identify the component of CA involved in the anti-inflammatory effects and isolated SA as the main compound using UHPLC-MS/MS analysis (Figure 5A). Consistent with the previous report, SA was isolated at a 5.54 retention time (RT) [27]. We examined the effect of SA on the mRNA expression of LPS-induced IL-6 and IL-1β in RAW264.7 macrophages. As shown in Figure 5B, LPS-induced increases in IL-6 and IL-1β mRNA expression levels were effectively inhibited in a dose-dependent manner by SA pretreatment. The inhibitory effect of SA was consistently observed on IL-1β protein expression (Figure 5C). Furthermore, SA effectively inhibited LPS-induced IκB phosphorylation in RAW 264.7 cells (Figure 5D). We also analyzed whether SA induces the expression of RCAN1. As shown in Figure 5E, SA treatment caused an increase in RCAN1 mRNA levels in RAW264.7 cells. Taken together, these results indicate that SA mediates the CA-induced anti-inflammatory signaling pathways by inducing RCAN1 expression (Figure 6). 

## 4. Discussion

CA is a perennial climber that grows at the foot of the mountain and blooms from May to June. The stem stretches upward, and the entire stem has dense, soft hairs. The dried root of CA is a traditional medicine used to treat a variety of inflammatory diseases, including abscesses, hectic fevers, and urinary infections [12,13]. Recently, a number of studies have reported that CA has anti-allergic, anti-acetylcholine esterase, and anti-parasitic activities [28,29]. CA also shows anti-tumor activities by inducing pro-apoptotic protein expression [27]. Although the effects of CA on immunosuppression have been reported, the signaling mediators and detailed underlying mechanisms are unclear. In the present study, we report the effect of CA as an inducer of RCAN1 and that RCAN1 expression is required for the CA-mediated anti-inflammatory effects in AD.

Several previous studies support our idea that RCAN1 acts as a mediator in anti-inflammatory signaling pathways. RCAN1-deficient mice displayed increased mortality following infection with bacterial pathogens, which is associated with higher levels of pro-inflammatory cytokines [30]. The IL-6 and TNF-α levels were higher BMMCs isolated from RCAN1-deficient mice than those from control mice following stem cell factor (SCF) stimulation [10]. In another report, RCAN1 deficiency caused an increase in the phosphorylation of IκBα and the transcriptional activity of NF-κB in the MyD88-dependent pathway [31]. In macrophages, RCAN1 is induced in response to pyrrolidine dithiocarbamate (PDTC) and thereby exerts an immunosuppressive effect [9]. In accordance with these reports, the overexpression of RCAN1 using adenovirus inhibited NF-κB and suppressed xenografted tumor growth in a severe combined immunodeficiency (SCID) model [32].

Because RCAN1 is identified as a direct calcineurin binding partner, the role of RCAN1 in the CA-mediated amelioration of AD symptoms appears to be related to calcineurin function. In support of this idea, calcineurin inhibitors such as cyclosporin A and FK506 have been widely used as remedies in the treatment of AD to prevent itching. When considering that these calcineurin inhibitors are difficult to apply for a long time due to their side effects, it is advantageous to apply a component derived from natural extracts, such as CA, which has fewer side effects to various inflammatory diseases.

In the previous study, the chemical composition of the CA extract was reported [27]. In the study by Son et al., sibiricose A1 (C_23_H_32_O_15_), 1-O-b-D-glucopyranosyl sinapic acid (C_17_H_22_O_10_), and sibiricose A4 (C_34_H_42_O_19_) were identified in CA alcohol extract [27]. Consistent with this report, the presence of SA in our CA extract was identified using UHPLC-MS/MS analysis. SA is found in natural herbs and has been reported to exert an anti-inflammatory function on diverse cells. SA is an orally available phytochemical found in cereals, fruits, vegetables, and oilseed crops [15]. SA has been tested against various diseases such as infections, cancer, diabetes, neurodegeneration, and anxiety [15]. SA alleviated the progression of osteoarthritis (OA) by reducing the levels of IL-1β, IL-6, prostaglandin E2 (PGE2), nitric oxide (NO), and TNF-α in vitro [33]. SA reduced inflammation, oxidative stress, and apoptosis by inhibiting NF-kB in cisplatin-induced nephrotoxicity [34]. The inhibition of inflammation and oxidative stress also has been shown in vascular endothelial cells [35]. In addition to these reports, our study suggests the anti-inflammatory effect of CA extract and its component, SA, in inflammatory signaling in Raw264.7 macrophages by inducing RCAN1 expression. The role of RCAN1 as an anti-inflammatory mediator was further demonstrated with RCAN1-knockout AD mice. Thus, our results reveal RCAN1 as a novel endogenous target that mediates the anti-inflammatory effect of CA and suggest the pharmaceutical potential of CA for the treatment of AD.

## Data Availability

Not applicable.

## Abbreviations

CA	*Cynanchi atrati*.
NF-κB	Nuclear factor-kappa B.
AD	Atopic dermatitis.
RCAN1	Regulator of calcineurin 1.
SA	Sinapic acid.
